# Construction of *Bordetella pertussis *strains with enhanced production of genetically-inactivated Pertussis Toxin and Pertactin by unmarked allelic exchange

**DOI:** 10.1186/1471-2180-12-61

**Published:** 2012-04-23

**Authors:** Wasin Buasri, Attawut Impoolsup, Chuenchit Boonchird, Anocha Luengchaichawange, Pannipa Prompiboon, Jean Petre, Watanalai Panbangred

**Affiliations:** 1 Department of Biotechnology, Mahidol University, 272 Rama 6 Road, Ratchathewi, Bangkok 10400, Thailand; 2 Bionet-Asia Co. Ltd., Hi-Tech Industrial Estate, 81 Moo 1, Baan-Lane, Bang Pa-In, Ayutthaya 13160, Thailand; 3 Mahidol University-Osaka University Collaborative Research Center for Bioscience and Biotechnology (MU-OU:CRC), Faculty of Science, Mahidol University, 272 Rama 6 Road, Ratchathewi, Bangkok 10400, Thailand

## Abstract

**Background:**

Acellular Pertussis vaccines against whooping cough caused by *Bordetella pertussis *present a much-improved safety profile compared to the original vaccine of killed whole cells. The principal antigen of acellular Pertussis vaccine, Pertussis Toxin (PT), must be chemically inactivated to obtain the corresponding toxoid (PTd). This process, however, results in extensive denaturation of the antigen. The development of acellular Pertussis vaccines containing PTd or recombinant PT (rPT) with inactivated S1, Filamentous Hemagglutinin (FHA), and Pertactin (PRN) has shown that the yield of PRN was limiting, whereas FHA was overproduced. To improve antigen yields and process economics, we have constructed strains of *Bordetella pertussis *that produce enhanced levels of both rPT and PRN.

**Results:**

Three recombinant strains of *Bordetella pertussis *were obtained by homologous recombination using an allelic exchange vector, pSS4245. In the first construct, the segment encoding PT subunit S1 was replaced by two mutations (R9K and E129G) that removed PT toxicity and Bp-WWC strain was obtained. In the second construct, a second copy of the whole cluster of PT structural genes containing the above mutations was inserted elsewhere into the chromosome of Bp-WWC and the Bp-WWD strain was obtained. This strain generated increased amounts of rPT (3.77 ± 0.53 μg/mL) compared to Bp-WWC (2.61 ± 0.16 μg/mL) and wild type strain (2.2 μg/mL). In the third construct, a second copy of the *prn *gene was inserted into the chromosome of Bp-WWD to obtain Bp-WWE. Strain Bp-WWE produced PRN at 4.18 ± 1.02 μg/mL in the cell extract which was about two-fold higher than Bp-WWC (2.48 ± 0.10 μg/mL) and Bp-WWD (2.31 ± 0.17 μg/mL). Purified PTd from Bp-WWD at 0.8-1.6 μg/well did not show any toxicity against Chinese hamster ovary (CHO) cell whereas purified PT from WT demonstrated a cell clustering endpoint at 2.6 pg/well.

**Conclusions:**

We have constructed *Bordetella pertussis *strains expressing increased amounts of the antigens, rPT or rPT and PRN. Expression of the third antigen, FHA was unchanged (always in excess). These strains will be useful for the manufacture of affordable acellular Pertussis vaccines.

## Background

Pertussis or whooping cough is a severe respiratory disease resulting from colonisation of the upper respiratory tract by the causative organism *Bordetella pertussis *[1]. Vaccines have been available for decades, comprising killed whole cells of *B. pertussis *that are chemically detoxified and formulated with Diphtheria and Tetanus antigens. They are administered as a trivalent Diphtheria-Tetanus-Pertussis combination, or in newer combinations with HBV and Hib, providing additional immunity against Hepatitis B and *Haemophilus influenzae *type b invasive disease, respectively [2]. The use of whole-cell Pertussis vaccines has been reduced, discouraged, or even banned in a few countries, due to the whole-cell vaccine's questionable safety profile, resulting from high level of endotoxin and other bacterial toxins associated with killed whole cells [3,4].

Acellular Pertussis vaccines (so-called because they do not contain whole cells but only partially- or extensively-purified bacterial antigens), were introduced in Japan in 1981 [5]. The higher purity of the component antigens in acellular Pertussis vaccines provided an improved clinical safety profile. These vaccines were introduced in the mid 90 s in other industrialized countries after extensive clinical trials that demonstrated their safety and efficacy [6]. A broader introduction by the WHO into the Expanded Program of Immunization was, however, hampered by the significantly higher cost of acellular Pertussis vaccines.

A major virulence factor of *B. pertussis *is Pertussis Toxin (PT) [7,8] and pertussis toxoid (PTd) is still the principal antigen in acellular vaccines [8]. Unlike Diphtheria and Tetanus toxins (that can be inactivated by simple treatment with formaldehyde), PT proved more difficult to be inactivated by chemical means [9]. At present, different inactivation processes are in use for commercial manufacture of acellular Pertussis vaccines. Unfortunately, all of them cause extensive denaturation of PT by their chemical treatments.

Two candidate vaccines have been tested using a genetically-inactivated toxin (rPT) [10-12] and one of these candidates was included in a field efficacy trial [11,12]. This vaccine was obtained by introducing two mutations into the catalytic subunit S1 of PT, causing abolition of the enzymatic activity of S1 and thus providing complete absence of toxicity of native PT. This vaccine was formulated with 5 μg rPT, 2.5 μg FHA and 2.5 μg PRN and was compared with another vaccine manufactured using classical chemical inactivation, comprising 25 μg PTd, 25 μg FHA and 8 μg PRN. The two vaccines had identical safety and efficacy results in this trial [13]. It was understood that the efficacy obtained with a lower dose of rPT and the other antigens was a result of using native antigens that included native FHA and PRN as the latter also required chemical treatment to inactivate residual traces of toxin when the antigens were derived from wild type *B. pertussis*.

Unfortunately, the vaccine described above, containing rPT, is not currently available due to unresolved intellectual property issues at the time of planned commercial introduction. Nevertheless, it is clear that the genetically-engineered approach to detoxification of Pertussis vaccine antigens is an essential element for the design of affordable acellular Pertussis vaccines, as intellectual property rights are expiring.

The vaccines referred to above contained three purified antigens derived from *B. pertussis *cultures: PTd or rPT, FHA and PRN. PT and even more so PRN are limiting antigens in *B. pertussis *cultures, while FHA is naturally overproduced. Alternative expression systems exist for increasing level of limiting *B. pertussis *vaccine antigens. PRN was expressed in high yield from recombinant *Escherichia coli *or from the recombinant yeast, *Pichia pastoris *[14,15]. PT subunits were expressed in *E. coli*, but unfortunately these failed to assemble into the mature toxin and were insufficiently immunogenic to be considered as potential vaccine candidates [16]. It is now understood that assembly and secretion of the mature toxin requires several auxiliary genes that were discovered more recently, and these genes are part of the *ptl *section of the *ptx-ptl *operon [17].

In this publication, we report the construction of recombinant *B. pertussis *strains expressing increased levels of rPT or rPT and PRN. These strains were generated by a multiple allelic- exchange process: insertion of the mutations that abolish the catalytic activity of subunit S1, insertion of a second copy of the *ptx *cluster of the five PT structural genes of the *ptx-ptl *operon with their promoter and terminator into an abandoned gene elsewhere on the chromosome, then insertion of a second copy of the *prn *gene into a second inactive gene locus. The organization of *ptl *auxiliary genes present in the *ptx-ptl *operon was not modified. Enhanced production of rPT and PRN by manipulation of gene copy number has been largely used with multi-copy plasmid vectors and reported to enhance the production of bacterial toxins [18,19], in particular PT [20]. However, genes tandemly repeated in this way may have significantly negative consequences on strain genetic stability in a GMP-regulated, vaccine-manufacturing environment. In addition, PRN expression could also be increased by manipulation of the PRN promoter [21].

The allelic-exchange vectors used in earlier *B. pertussis *recombinant strains require mutations on the chromosome, particularly the mutation affecting *rpsL *that results from selection of spontaneous streptomycin-resistant mutants as required in earlier allelic-exchange procedures [22]. Such mutations affecting housekeeping genes may impair virulence, hence the expression of virulence factors including PT, FHA and PRN. In contrary, pSS4245 used in this study harbours streptomycin resistant gene from Tn5 which is functional in *B. pertussis *but not in *E. coli*, hence streptomycin was used to select against *E. coli *donor cell and I-SceI nuclease activity in the plasmid was then functioned as the counter selectable marker in the recombinant *B. pertussis *through subsequent homologous recombination and does not require or leave auxiliary mutations. The strains reported here produce unaltered levels of the other antigens in particular FHA. These constructs will prove useful for the manufacture of affordable human acellular Pertussis vaccines.

## Results

### Mutation of the S1 gene in the B. Pertussis chromosome

**Figure 1:**
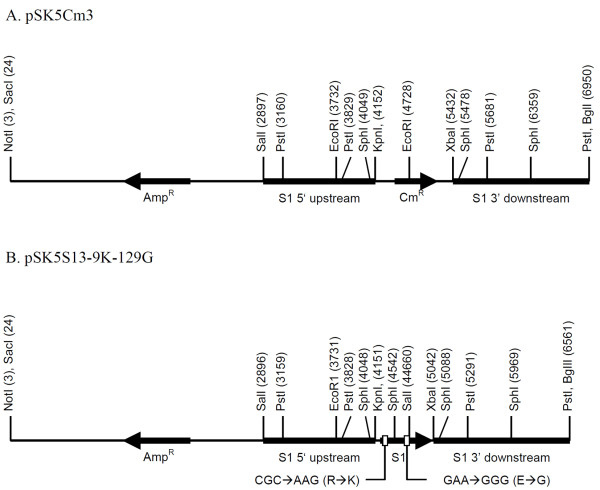
**Vectors for the construction of a modified S1 gene into the allelic-exchange vector pSS4245**. A: Allelic-exchange element for replacing the S1 gene by a chloramphenicol resistance cassette, inserted between the S1 flanging regions. B: Allelic-exchange element for returning the modified S1 gene into its exact location in the *ptx*-*ptl *operon. To obtain the allelic exchange, these vectors were linearized and inserted into pSS4245, which was then introduced into *B. pertussis *by conjugative transfer from *E. coli *SM10

**Figure 2:**
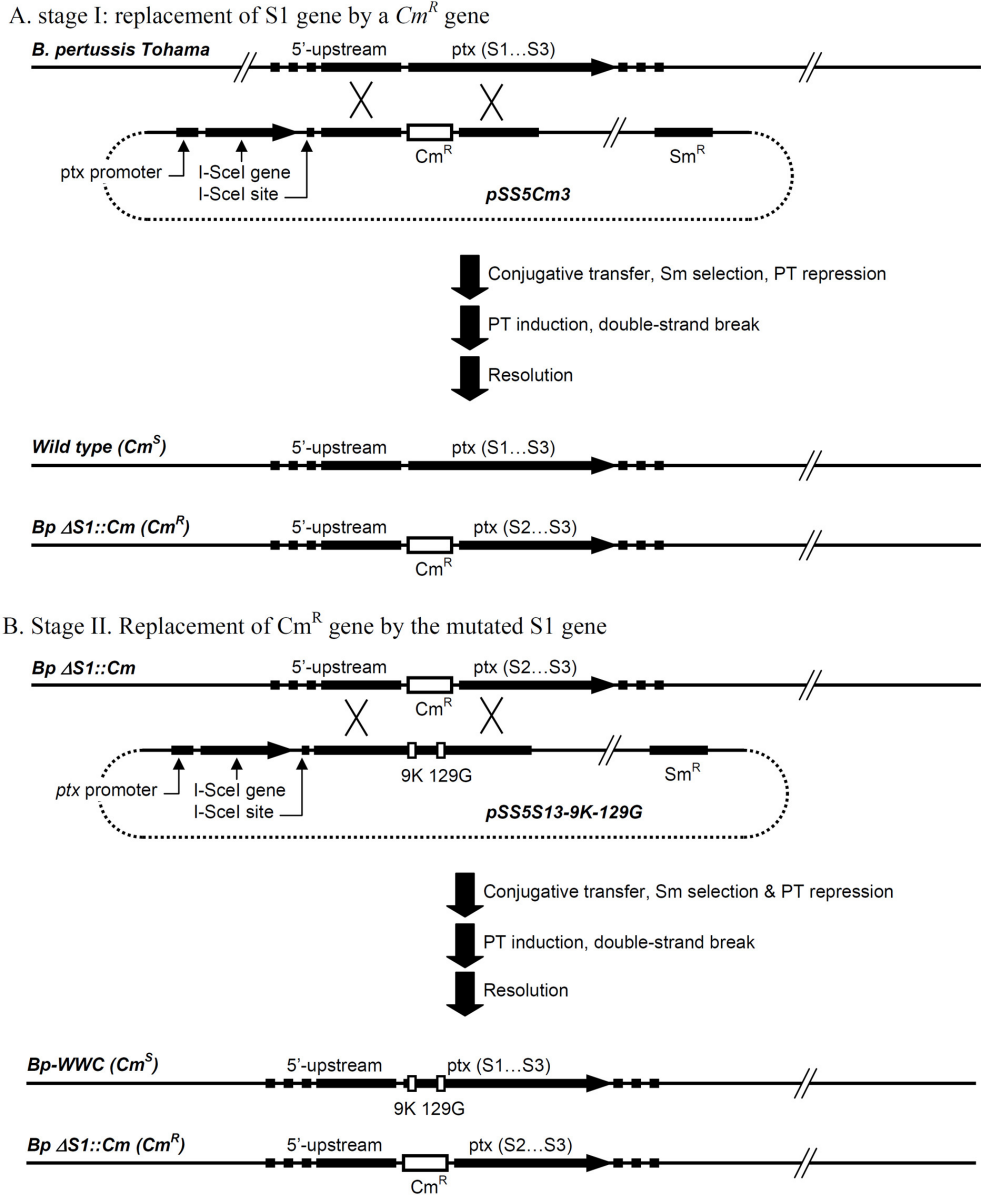
**Allelic-exchange procedure**. A: Double recombination events leading to the replacement of the S1 gene by a chloramphenicol resistance marker. B: Double recombination events leading to the re-insertion of the modified S1 gene in its original location.

To introduce the two mutations R9K and E129G into the S1 subunit, a two-stage approach was used to avoid the possibility of recombination in the region between the two mutations that would cause the loss of one of the mutations. This approach also allowed selection of the desired colonies by simple replica plating on selective media. Firstly, two *E. coli *vectors were constructed in pBluescript II SK + where the wild-type *S1 *gene was replaced by a chloramphenicol resistance gene (*Cm^R^*) (Figure 1A) or by a modified *S1 *gene including the desired mutations (Figure 1B); both flanked by 1.2 and 1.5 kb of the *S1 *upstream and downstream regions, respectively. These vectors were then processed and their inserts were introduced into pSS4245. These derivatives were transferred into *E. coli *SM10 for conjugative transfer and allelic exchange into *B. pertussis *strain Tohama. The plasmid pSS5Cm3 generated a replacement of the *S1 *gene by the *Cm^R ^*marker (Figure 2A). The plasmid pSS5S13-9 K-129 G restored the *S1 *gene into its original location, now with the two desired mutations (Figure 2B). After selection of isolates on selective media, integration of the *Cm^R ^*and modified *S1 *genes at the expected position was confirmed by PCR amplification (data not shown). The integration of the mutated *S1 *gene at the designated position was confirmed by PCR with specific primers that could hybridize the upstream 5 and 3 prime downstream flanking regions and internally in the *S1 *gene (data not shown). The mutations in the *S1 *gene of the clone selected for further manipulation was confirmed by DNA sequencing. The new strain was designated as Bp-WWC.

### Insertion of a second integration site for a second set of PT structural genes

**Figure 3:**
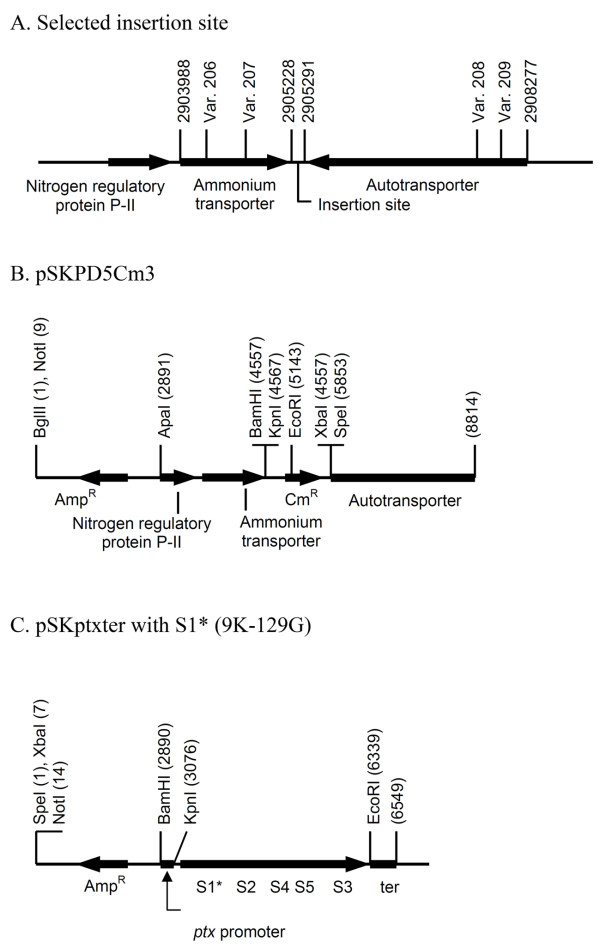
**Vectors for the insertion of a second copy of the *ptx *operon into the *B. pertussis *chromosome**. A: The insertion site for a second copy of the *ptx *operon was selected between two abandoned genes, each carrying two frameshift mutations. B: Allelic-exchange elements used to insert a chloramphenicol marker into the selected site. C: Schematic structure of the *ptx *operon with its original promoter. The *ptx-ptl *terminator was cloned and inserted downstream of the S3 gene. This cluster was finally integrated into the SS4245 derivative to replace the chloramphenicol marker and generate the second allelic-exchange event to insert the second copy of the PT structural genes.

Initial attempts to increase PT expression by inserting the whole *ptx-ptl *operon into a multi-copy plasmid compatible with *B. pertussis *failed to deliver useful strains suggesting that the over-expression of PT is potentially toxic and must remain within certain limits to obtain viable strains. In order to increase the PT toxin yield, a second set of PT structural genes was introduced into the Bp-WWC chromosome. To identify an insertion target site, the sequence of the *B. pertussis *Tohama genome (accession number NC_002929) was scanned and many pseudogenes were identified. The DNA sequence (posn. 2905288) between a putative ammonium transporter gene and a putative auto-transporter gene was selected for insertion (posn. 2903988-2905228 and 2905291-2908277). These genes each carry frameshift mutations which ruin their functionality (Figure 3A). The general strategy outlined in the preceding section was followed. First, the *E. coli *vector pSKPD5Cm3 was constructed by inserting the *Cm^R ^*gene within the regions flanking the selected integration site (Figure 3B). After insertion of the sequences of interest into pSS4245, allelic exchange was selected by the *Cm^R ^*marker. Integration of the *Cm^R ^*gene at the designated position was confirmed by PCR (data not shown). In the second vector, five PT structural genes with mutated *S1 *were inserted between the *ptx*-*ptl *operon promoter and terminator (following the *S3 *gene) to generate the vector pSKptxter (Figure 3C). Allelic exchange into the selected target integration inserted a second copy of the functional cluster of the PT structural genes into Bp-WWC strain. The new strain was designated as Bp-WWD. This strain harboured two copies of *ptx *operon with mutated *S1 *gene. The result of integration was verified by amplification of the upstream, downstream, and internal regions of the *ptx *operon, that all showed the expected integration without disruption of the regions where recombination had occurred.

### Sequencing of the S1 gene and identification of the R9K and E129G mutations

**Figure 4:**
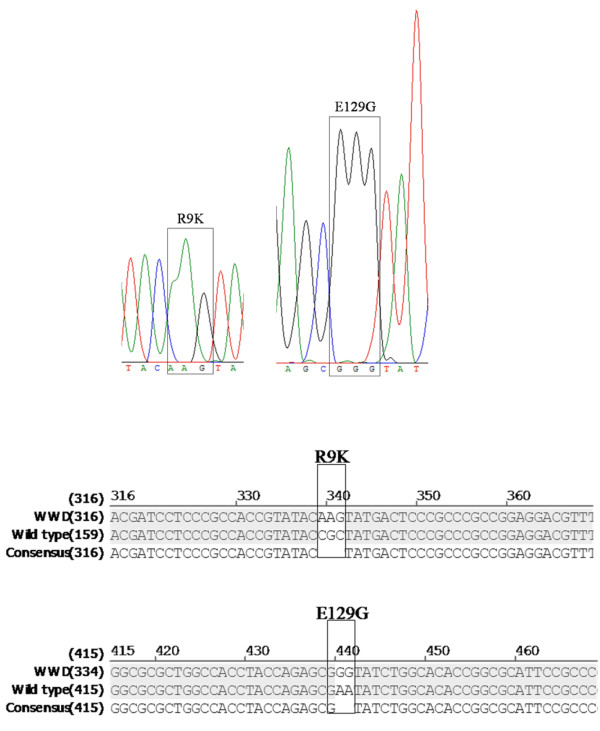
**Identification of the R9K and E129G mutations in Bp-WWC and Bp-WWD**. Raw sequence data around the mutations are shown for strain Bp-WWD that has two copies of the PT structural cluster. The corresponding sequence alignments are shown for *B. pertussis *Tohama (consensus sequence) and derivatives Bp-WWC and Bp-WWD.

Automated sequencing was applied to confirm the presence of the desired mutations. In the case of strain Bp-WWD that has two integrated copies of the *S1 *gene, PCR amplification yields, in principle, a mix of the copies of the two genes. An unexpected point mutation in one of the inserts would appear as a double-nucleotide assignment at the corresponding position. The single peak of fluorescence signal at the R9K and E129G positions indicated the correct sequence on Bp-WWC and that of the two copies of *S1 *in Bp-WWD had identical mutations. The sequence around the two desired mutations is reported in Figure 4 that shows the sequencing records for strain Bp-WWD and the sequence alignments for wild-type Tohama, Bp-WWC and Bp-WWD.

### Insertion of a second copy of the prn genes into the Bp-WWD strain

**Figure 5:**
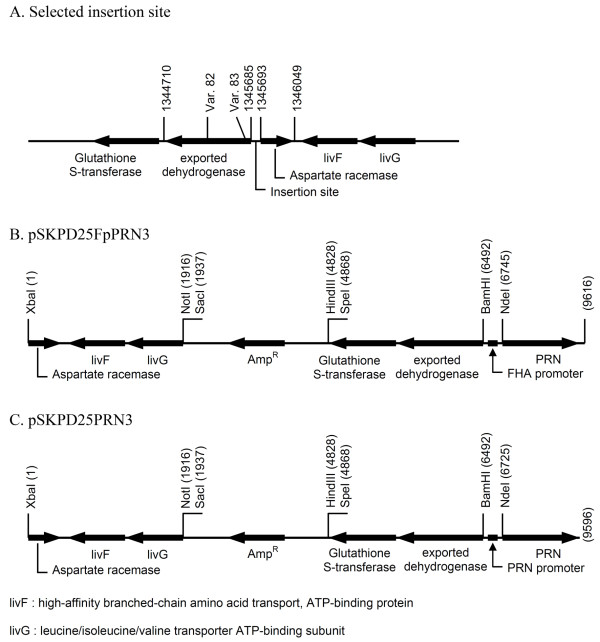
**Vectors for the insertion of a second copy of the *prn *gene into the *B. pertussis *chromosome**. **A**: The insertion site for a second copy of the *prn *gene was selected between two abandoned genes carrying frameshift mutations and a deletion. **B**: Schematic structure of the *prn *gene under control of *fha *promoter and flanking with target integration site. **C**: Schematic structure of the *prn *gene under control of its own promoter and flanking with target integration site.

Due to the low level of PRN expression, a second copy of the *prn *structural gene (under control of the 246 bp *fha *promoter and its own terminator) was introduced into the Bp-WWD chromosome (posn. 1345693) between the two pseudogenes of putative exported dehydrogenase (posn. 1344710-1345685) and a putative aspartate racemase (posn. 1345693-1346049) (Figure 5A). The pSKPD2Cm3 *E. coli *vector was constructed where the *Cm^R ^*gene was inserted between the upstream and downstream regions flanking the selected insertion site. Another vector was constructed using the same flanking regions and the *prn *gene under control of the *fha *promoter (Figure 5B). After insertion of the *Cm^R ^*marker in the desired location, the *Cm^R ^*gene was replaced by the *prn *functional block using the usual allelic-exchange selection and screening procedures.

The *B. pertussis *strains isolated from this construction exercise did not express PRN and the expression level of the other (FHA, PT and hemolysin) antigens was not detectable (data not shown). It was tentatively concluded that the PRN product is toxic if overproduced under control of the stronger *fha *promoter and only escape mutants having lost the capacity to produce PRN or all virulence factors were viable. It was, therefore, decided to introduce the natural *prn *promoter in place of the *fha *promoter. The plasmid pSKPD25FpPRN3 was used to replace the *fha *promoter by the original *prn *promoter to generate a functional cassette with its own natural promoter and terminator (Figure 5C). This functional cassette was inserted at the selected site by the usual allelic-exchange procedure to obtain a strain with a second non-tandemly-repeated copy of the *prn *gene under control of its own promoter. The expected insertion was confirmed by PCR amplification with primers binding to the flanking regions internally in the *prn *gene. This strain was normally viable and was designated as Bp-WWE.

### Genetic stability of PT and PRN constructs in Bp-WWE

The strain Bp-WWE was cultured and serially sub-cultured in Modified Stainer-Scholte (MSS) medium to reach approximately 50 generations. The last culture was diluted and plated onto MSS agar. Thirty isolated colonies were randomly picked, and analyzed for their *S1 *and *prn *genes by PCR (data not shown). The results showed that all colonies contained two copies of *S1 *and *prn *genes at the expected positions.

### Expression of PT, FHA and PRN in shake flasks

The production of PT and FHA in shake flask cultures was analyzed by ELISA. Shake flask cultures were all performed in MSS medium containing heptakis(2,6-O-dimethyl)β-cyclodextrin [23,24]. At 36 h, the production of PT was about doubled in strain Bp-WWD (3.77 ± 0.53 μg/mL), compared with Bp-WWC (2.61 ± 0.16 μg/mL) and wild-type Tohama (2.2 μg/mL) (Table 1), demonstrating that the level of PT expression was a function of the number of copies of the structural gene cluster. FHA in all three recombinant strains was about the same (Table 1). The production of PRN in shake flask cultures of Bp-WWC, Bp-WWD and Bp-WWE in MSS medium was analyzed by densitometry analysis of Western blot results. PRN amount in the clarified culture supernatants and extract of the separated cells at 60°C was assayed. The amount of PRN in cell extract of Bp-WWC and Bp-WWD was similar (2.48 ± 0.10 and 2.31 ± 0.17 μg/mL, respectively). A two-fold increase was found in Bp-WWE (4.18 ± 1.02 μg/mL), again showing a good correlation of the level of *prn *expression to the gene copy number. In all three recombinant strains, the fraction of PRN found in the supernatant fraction in these flask cultures was small or negligible (less than 0.1 μg/mL, data not shown).

**Table 1:** PT, FHA and PRN production by strains Bp-WWC and Bp-WWD and Bp-WWE

Strain	PT (μg/mL)	FHA (μg/mL)	PRN (μg/mL)**
Tohama wt	2.2	ND*	ND*

Bp-WWC	2.61 ± 0.16	17.75 ± 3.30	2.48 ± 0.10

Bp-WWD	3.77 ± 0.53	14.33 ± 0.50	2.31 ± 0.17

Bp-WWE	4.49 ± 0.83	17.08 ± 2.21	4.18 ± 1.02

*ND = Not determined **The amount in cell extract

The values were the mean of 3 independent experiments with standard deviation except the data for PT of Tohama WT was obtained from two independent experiments

### Assessment of PT inactivation

**Figure 6:**
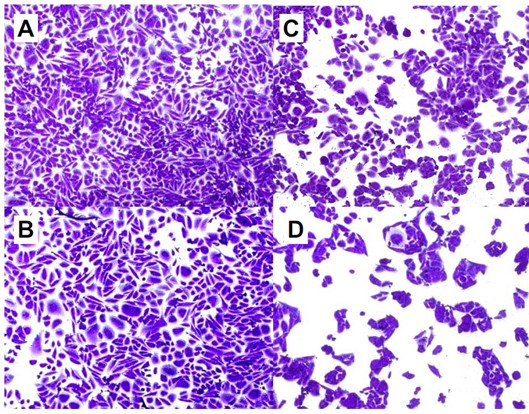
**CHO-cell clustering test**. The cells were grown to near confluence then dilutions of PT were added and the clustering was scored after 2 days. A: 800 ng PT (strain Bp-WWC). B: Control, no PT added. C: 2.6 pg wt PT (strain Tohama) corresponding to the limit of detection. D. 43 pg wt PT (strain Tohama)

PT was purified from culture supernatants using a modification of the process published by Ozcengiz [25] where the initial ammonium sulphate precipitation was replaced by ligand exchange chromatography [26,27]. The toxicity of the PT toxin from wild type *B. pertussis *and Bp-WWC (genetically inactivated PT) was analysed and compared by the Chinese hamster ovary (CHO) cell clustering assay [28]. This assay has a much higher sensitivity than other functional assays reported for PT. The native toxin purified from strain *B. pertussis *Tohama demonstrated a clustering endpoint at 2.6 pg per well. The genetically-inactivated PT did not promote clustering at the highest concentrations of 0.8-1.6 μg per sample obtained in this test (Figure 6). This assay can, therefore, detect toxicity reduction by a factor of 5 × 10^5 ^to 1 × 10^6^, despite limitations imposed by the low solubility of PT. This result demonstrated that PT toxin purified from Bp-WWC was successfully inactivated by insertion of five nucleotide replacements resulting in two amino acid replacements in the PT subunit S1.

## Discussion

Unmarked gene insertion and replacement were successful, using pSS4245 as vector in *B. pertussis*. After a second homologous recombination to excise the plasmid, no antibiotic gene marker nor any scars were left in the chromosome when compared with the cre-lox system [29] or earlier allelic-exchange procedures used in *Bordetella *[22]. Overproduction of genetically-deactivated PT toxin was reported in 1992 [20] by using tandem repeats of *ptx *genes or another copy inserted into the *fha *gene. The resulting recombinant *B. pertussis *strain overproduced PT up to 80 mg/L. Tandemly-repeated genes are a known potential cause of genetic instability. For this reason, the genome sequence of *B. pertussis *was scanned to look for suitable integration sites. The DNA position between two terminators of pseudo-genes (putative ammonium transporter and putative auto-transporter genes) was selected as integration sites for the *ptx *cluster. The copy number for the PT structural cluster was limited to two, as overproduction of these virulence factors places a burden on cell metabolism, resulting in slower growth and potentially genetic instability, as shown by preliminary results.

Over-expression of *prn *gene by the *fha *promoter to drive higher expression was apparently toxic to growth of *B. pertussis*, possibly in resulting from higher PT expression. Our results showed that replacement of the *prn *promoter with a stronger one did not provide increased *prn *expression [21]. Therefore, increasing the gene copy number under the control of the native *prn *promoter was the approach selected. The *fha *promoter of the second gene copy was replaced by the native *prn *promoter to generate a strain with a second copy of the *prn *gene and its native promoter inserted into another location on the chromosome. The toxicity of PRN to the host cell was also reported in *E. coli *[30]. The *fha *promoter was then replaced by the native *prn *promoter, then the resulting strain exhibited normal growth in shake flasks and expressed twice the amount of PRN. The distribution of PRN between culture supernatant and cell extract was modified - a larger fraction of total PRN was found in the supernatant although in shake flasks, the quantities of PRN spontaneously released into the supernatant were minimal. The presence of either two copies of mutated *PT *gene alone or together with two copies of *prn *in WWC, WWD or WWE did not show any genetic instability as evidenced by serial-subculture experiment. All recombinant strains showed the presence of two copies of corresponding genes and corresponding amount of PT and PRN. Hence, homologous recombination among the homologous copies was not so far found in these strains.

Although bacterial growth in shake-flask culture is limited due to rapid pH rise and intoxication from release of ammonia by metabolism of the glutamate carbon source [31], shake-flask culture provides a useful indication of the strain's potential under optimized fermentor conditions. The construction of stable strains with enhanced expression of PT (Bp-WWD) or of the two limiting antigens PT and PRN (Bp-WWE) was demonstrated. With enhanced production of PT alone, Bp-WWD could not generate sufficient quantities of PRN, therefore in this case, the use of an independent supply of PRN in recombinant *E. coli *or *P. pastoris *would be required. As the expression level of both PT and PRN has been equally increased in strain Bp-WWE, it would be expected that matching quantities of the two antigens would also be obtained in higher-density cultures, thereby simplifying vaccine manufacturing operations.

## Conclusions

*B. pertussis *strains that contains genetically-inactivated S1::R9K-E129G subunits of PT were constructed without leaving any markers or scars in their chromosomes. An about two-fold increase in expression of PT toxin was found in shake flasks by integrating the 5 structural genes (*ptx *with *S1 *mutated) under the control of the *ptx-ptl *operon promoter and terminator between two pseudo-genes on the chromosome. The presence of detoxified PT was confirmed by the CHO cell clustering assay. In addition, PRN production was increased by integration of a second copy of the *prn *gene between other pseudo-genes located elsewhere on the chromosome. The strains were found to be genetically stable in shake flask sub-cultures at higher generation numbers than would be required to reach large-scale fermentations (> 1,000 L). These recombinant strains, in particular, strain Bp-WWE (where the ratio of expression of PT and PRN antigens matches the composition of commercial Pertussis vaccines), should enable production of affordable acellular Pertussis vaccines. The lower Cost of Goods (CoG) is provided by the lower dose of native antigens required for adequate immunogenicity and the higher productivity the two limiting antigens PT and PRN.

## Methods

### Bacterial strains, plasmids and culture conditions

All chemicals and reagents used in this study were either molecular biology or analytical grade. Chemicals were purchased from Merck (Germany) and Sigma (USA). Bacterial culture media were obtained from Difco (USA) and Merck. Restriction and modifying enzymes were purchased from New England Biolabs (USA).

*E. coli *DH5α (Invitrogen, USA) was used as a cloning host. This strain was grown at 37°C in Luria Bertani (LB) medium. The *E. coli *DH5α transformants were grown in LB medium supplemented with appropriate antibiotics: amplicillin (50 μg/mL) or chloramphenicol (15 μg/mL). *E. coli *SM10 and pSS4245 were obtained from Dr. Earle S. Stibitz and used as a conjugative donor strain and an allelic exchange vector, respectively. This strain was grown at 37°C in LB medium supplemented with kanamycin (50 μg/mL). The *E. coli *SM10 transformants were grown in LB medium supplemented with kanamycin (50 μg/mL), amplicillin (50 μg/mL) and neomycin (10 μg/mL). *B. pertussis *Tohama was obtained from ATCC (BAA-589). *B. pertussis *strains were grown at 35°C on Bordet-Gengou (BG) agar or MSS medium [32]. One liter of the MSS medium contained 10.7 g of monosodium glutamate, 0.24 g of L-proline, 2.5 g of NaCl, 0.5 g of KH_2_PO_4_, 0.2 g of KCl, 0.1 g of MgCl_2_·6H_2_O, 0.02 g of CaCl_2_·2H_2_O, 6.1 g of Tris base, 10 g of casamino acids 0.01 g of FeSO_4_·7H_2_O, 0.04 g of L-cysteine, 0.1 g of glutathione, 0.02 g of ascorbic acid, 0.004 g of niacin and 1 g of dimethyl-β-cyclodextrin. Plasmid pBluescript II SK + and pACYC184 were obtained from Stratagene (USA) and New England Biolabs (USA), respectively.

### Cloning of S1 flanking regions and insertion of a chloramphenicol gene

The chromosomal DNA of *B. pertussis *strain Tohama was used as source material. The upstream region of the S1 gene was amplified by PCR using the 5'F-PT-SalI and 5'R-PT-MCS primers. The latter contained *Kpn*I, *Xba*I, *Bgl*II and *Not*I sites. The amplification product was recovered from agarose gel and purified by QIAEX II Extraction kit (Qiagen, Germany). The 1287 bp amplification product was digested with *Sal*I and *Not*I and cloned into the *E. coli *vector pSKΔKpnI digested with the same enzymes. pSKΔKpnI was a derivative of pBluescript II SK + where the *Kpn*I site was removed by digestion, trimming 3' protruding end by the Klenow enzyme, and re-circularization. The resulting construct was transformed by heat shock into competent cells of *E. coli *DH5α and designated as pSK5'. The downstream region was likewise obtained by amplification with the 3'F-PT-XbaI and 3'R-PT-BglII primers. The 1531 bp product was digested with *Xba*I and *Bgl*II and the recovered fragment inserted into pSK5' digested with the same enzymes to obtain pSK53.

The *Cm^R ^*gene was obtained from plasmid pACYC184. The gene was amplified using the primers CmF-KpnI and CmR-XbaI. The 1295 bp PCR product was purified and digested with *Kpn*I and *Xba*I and inserted into pSK53 cut with the same enzymes. The resulting plasmid was designated as pSK5Cm3. This plasmid incorporated the chloramphenicol resistance gene flanked by the 5'-upstream and 3'-downstream regions of the *S1 *gene (Figure 1A).

### Exchange of the S1 gene by homologous recombination

To perform the allelic exchange, vector pSS4245 [33] was used. Plasmid pSK5Cm3 was digested with *Sac*I and *Bgl*II and the recovered fragment ligated into pSS4245 cut with *Sac*I and *Bam*HI. After transformation into *E. coli *SM10, the resulting plasmid was designated as pSS5Cm3. Fresh cultures of *B. pertussis *strain Tohama (4 days on MSS-agar with 20 mM nicotinic acid) and of *E. coli *SM10 harbouring the vector (overnight on LB-agar with ampicillin, kanamycin and chloramphenicol) were scraped and mixed onto agar plates containing LB:MSS (1:1) with 20 mM nicotinic acid and 10 mM MgCl_2_. After 3 h-cultivation at 35°C, the mix was swabbed onto MSS with 20 mM nicotinic acid, 50 μg/mL streptomycin and 5 μg/mL chloramphenicol. Streptomycin and chloramphenicol were used to select against *E. coli *SM10 (donor cell) and *B. pertussis *(recipient cell). The swab growth was streaked onto MSS agar with 5 μg/mL chloramphenicol for the second recombination event. The resulting single colonies were tested by replica plating and a few colonies with the Sm^S ^and Cm^R ^phenotype were retained for further testing (Figure 2A). The integration of *Cm^R ^*gene at designed position was confirmed by PCR using the primers that specifically bind to the upstream 5' (5'F-int and 5'RCM-int primers) and 3' (3'FCM-int and 3'R-int primers) downstream flanking regions and internally in the *Cm^R ^*gene. From the PCR analysis, it was confirmed that the 5' and 3' flanking regions were present and the *Cm^R ^*gene had been inserted at the expected location in place of the S1 gene. These verifications also confirmed that the allelic-exchange process had not caused any alteration in the *S1 *flanking regions where recombination had taken place.

### Construction of a modified S1 gene

The *S1 *gene was cloned by PCR amplification and mutated by site-directed PCR mutagenesis. The primers S1F-PT-KpnI and S1R-PT-XbaI (Table 2) were used to amplify the gene from chromosomal DNA. The purified PCR product was digested with *Xba*I and *Kpn*I and the recovered 908 bp fragment was ligated into pSK53 cut with the same enzymes. After transformation and colony selection, the resulting plasmid was designated as pSK5S13.

**Table 2:** Primers used for construction

Name	Sequence
5'F-PT-SalI	GCG**GTCGAC**GGCGCGCAATGCGGCGCGGAC

5'R-PT-MCS	GGGG**GCGGCCGC**G**AGATCT**C**TCTAGA**C**GGTACC**ATCGCGCGACTTTGCGCCGAAGGA

3'F-PT-XbaI	CGT**TCTAGA**CCTGGCCCAGCCCCGCCCAAC

3'R-PT-BglII	GGC**AGATCT**GCAGTTCGAGCAGATCGCCGG

CmF-KpnI	CGC**GGTACC**TGATGTCCGGCGGTGCTTTTG

CmR-XbaI	AA**TCTAGA**TATCGTCAATTATTACCTCCAC

S1F-PT-KpnI	GAT**GGTACC**GGTCACCGTCCGGACCGTGCT

S1R-PT-XbaI	CAGG**TCTAGA**ACGAATACGCGATGCTTTCG

R-R9K	GGGCGGGAGTCATACTTGTATACGGTGGCGG

F-R9K	CCGCCACCGTATACAAGTATGACTCCCGCCC

F-E129G	CCACCTACCAGAGCGGGTATCTGGCACACCGG

R-E129G	CCGGTGTGCCAGATACCCGCTCTGGTAGGTGG

5'F-PD-ApaI	GGA**GGGCCC**ATGAAACTCGTCATCGCCATCATCAAGCCC

5'R-PD-MCS	TAC**GGTACCGGATCC**CGCATCGCAACAACGGGGTCATCGCGACCC

3'F-PD-MCS	CGT**TCTAGAACTAGT**CCGCTACCAGGTGTAGCGATAGCCCAGGTG

3'R-PD-BglII	TGT**AGATCT**CGGCGAGATACTTGCGTTTCGGCGTTGTCG

PtxF-BamHI	TTG**GGATCC**CAGCGCAGCCCTCCAACGCGCCATCC

PtxR-MCS	TCT**ACTAGT**AA**GAATTC**TCGCGGTATCCGTCAAGGAAAAACATGGAC

TerF-EcoRI	GCG**GAATTC**CGCCTGCCGCCTGCACGCAT

TerR-SpeI	TCC**ACTAGT**CAAGGGCATCGGGCGCCGGC

5'F-PD2-SpeI	CGC**ACTAGT**CTATTCCAGCGGCGGGTCGAAATGGC

5'R-PD2-MCS	CCCCAG**GCGGCCGC**TG**TCTAGA**GT**GGATCC**CAGGCCGATGCGTCCGCCGTGCAGGC

3'F-PD2-XbaI	ATC**TCTAGA**ATGGGCACCTCGGCCACGCTGGCGCTG

3'R-PD2-NotI	AAGTAT**GCGGCCGC**ATGAGCGAAACCCTGTTGAAAGTATC

CmF-BamHI	CGC**GGATCC**TGATGTCCGGCGGTGCTTTTG

FHAproF-BamHI	TCT**GGATCC**CTGCGCTGGCACCCGCGGCGGGCCG

FHAR-MCS	GCC**TCTAGA**TT**CATATG**ATTCCGACCAGCGAAGTGAAGTAAT

PRNF-NdeI	CTGGTCGG**CATATG**AACATGTCTCTGTCACGCATTG

PRNF	ATGAACATGTCTCTGTCACGCATTGTCAAGG

PRNR-XbaI	GCC**TCTAGA**GCCTGGAGACTGGCACCGGCCAAGC

PrnProF-BamHI	CGG**GGATCC**GCACCCTGGCCTGCGGGGCGGGACC

PRNProR-NdeI	AGACATGTT**CATATG**GATGCCAGGTGGAGAGCAGA

5'F-int	CTAGCGTTCGCATACCAAATCCTTGC

5'RCM-int	CCGTAATATCCAGCTGAACGGTCTGG

3'FCM-int	TCTGTGATGGCTTCCATGTCGGCAG

3'R-int	AGCATGTTGCGGTGTTCCCGGAATG

5'FPD-int	ATGACGGAAAGCCGCATGGGCATTGGGTCC

3'RPD-int	TTCGTACGTGTTCAGGTGCCGATTGCCGG

5'FPD2-int	TGGGCTGGCTGTTCTGGCACGAAACG

3'RPD2-int	TTCATCGAATCGGCGCTGATCCTGGC

PRNF-int	AGGTGCAGCCATACATCAAGGCCAGC

Sequences with bold letters are the recognition sites for various restriction enzymes used for each construction. Underlined sequences are the sequences of the new codons used for constructing mutant

Site-directed PCR mutagenesis used the internal F-R9K and R-R9K primers with the sequence mismatch CGC→AAG, causing the R9K substitution. The same procedure was applied to generate the second mutation using the internal mismatched primers F-E129G and R-E129G, to generate the sequence GAA→GGG, causing the E129G substitution.

The resulting fragment was digested with *Xba*I and *Kpn*I and inserted into pSK53 cut with the same enzymes to obtain plasmid pSK5S13-9 K-129 G (Figure 1B). This was digested with *Sac*I and *Bgl*II and the recovered fragment was ligated into pSS4245 cut with *Sac*I and *Bam*HI. After transformation into *E. coli *SM10, the resulting plasmid was designated as pSS5S13-9 K-129 G.

Allelic exchange to insert the modified *S1 *gene back into its original location in the *B. pertussis *chromosome was performed as above but without selection of the exconjugants by chloramphenicol. The desired strains in this case have lost this marker and therefore screening by replica plating was necessary to identify colonies with the desired phenotype Cm^S ^and Sm^S^. The resulting Tohama derivative was designated as Bp-WWC (Figure 2B). The integration of the *S1 *mutated gene at the designated position was confirmed by PCR with the specific primers. The primers could bind the upstream 5' (5'F-int and R-R9K primers), 3' (F-E129G and 3'R-int primers) downstream flanking regions, and inside the *S1 *gene.

### Insertion of a second set of the 5 PT structural genes

The sequences flanking the targeted insertion site (Figure 3A) were first cloned to obtain pSKPD5Cm3. The upstream 1688 bp fragment was amplified with the primers 5'F-PD-ApaI and 5'R-PD-MCS, digested with *Apa*I and *Kpn*I, and ligated into pSK5Cm3 cut with the same enzymes to yield pSKPD5'-Cm. The downstream 2980 bp fragment was amplified with the primers 3'F-PD-MCS and 3'R-PD-BglII, digested with *Xba*I and *Bgl*II, and ligated into pSKPD5'-Cm cut with the same enzymes. The resulting plasmid was designated as pSKPD5Cm3 (Figure 3B).

The conjugative construct was obtained by digesting this plasmid with *Not*I and *Bgl*II and ligation into pSS4245 which was digested with *Not*I and *Bam*HI, resulting in plasmid pSSPD53-Cm. Conjugative transfer and selection for Sm^S ^and Cm^R ^provided the desired *B. pertussis *derivative Bp-PD53-Cm, where the presence of the intact upstream, downstream, and Cm^R ^insert was confirmed by PCR amplification. The primers could bind the upstream 5' (5'FPD-int and 5'RCM-int primers), 3' (3'FCM-int and 3'RPD-int primers) downstream flanking regions, and inside the *Cm^R ^*gene.

A functional copy of the *ptx *operon with its promoter was generated by insertion of the *ptx-ptl *terminator next to the S3 gene. The five structural genes of PT (modified *S1, S2, S4, S5, and S3*) with its operon promoter were amplified from Bp-WWC DNA using the primers PtxF-BamHI and PtxR-MCS. The 3469 bp amplified product was digested with *Bam*HI and *Spe*I and the recovered fragment was ligated into pSKΔRI cut with the same enzymes to yield pSKptx. Plasmid pSKΔRI is a variant of pBluescript II SK + where the *Eco*RI site has been removed by digestion and filled-in with the Klenow enzyme and re-circularized.

The *ptx-ptl *operon terminator was then amplified with the TerF-EcoRI and TerR-SpeI primers. The 223 bp product was doubly digested with *Eco*RI and *Spe*I and ligated into pSKptx cut with the same enzymes. After transformation and colony selection, the resulting plasmid was designated as pSKptxter (Figure 3C). This plasmid was then doubly digested with *Bam*HI and *Spe*I and ligated into pSSPD5Cm3 cut with the same enzymes to yield the conjugative vector pSSPDptxter. Allelic exchange into Bp-PD53Cm was performed as described above with replica screening for Sm^S ^and Cm^S ^colonies to obtain the strain designated as Bp-WWD. The integration of *S1 *mutated gene at the designated position was confirmed by PCR with specific primers. The primers could bind the upstream 5' (5'FPD-int and R-R9K primers), 3' (F-E129G and 3'RPD-int primers) downstream flanking regions, and internal *S1 *gene.

### Insertion of a second copy of the prn structural gene

#### Integration of a chloramphenicol resistance gene into the target site selected for integrating a second copy of the PRN structural gene

A derivative of pBluescript SK + lacking the *Bam*HI site was constructed by digestion with the enzyme, filling-in with the Klenow enzyme, and ligation. The resulting plasmid was transformed into *E. coli *and designated as pSKΔH1.

The sequence of the *B. pertussis *Tohama strain was scanned and pseudo-genes were identified. The DNA sequence (posn. 1345693) between a putative exported dehydrogenase (posn. 1344710-1345685) and a putative aspartate racemase pseudo-gene (posn. 1345693-1346049) was selected as the insertion site. These two genes carried frameshift mutations and were not functional (Figure 5A). The 5'-upstream region to the targeted insertion site was amplified using primers carrying *Spe*I (5'F-PD2-SpeI) and a multilinker including *Bam*HI and *Not*I (5'R-PD2-MCS) restriction sites. The amplified product was isolated by gel electrophoresis and doubly digested with *Spe*I and *Not*I. The resulting fragment was ligated into a fragment of pSKΔH1 which was digested with the same enzymes. The resulting plasmid was transformed into *E. coli *and designated as pSKPD25. The 3'-downstream fragment was similarly amplified with primers carrying *Xba*I(3'F-PD2-XbaI) and *Not*I (3'R-PD2-NotI) restriction sites. After digestion with the same enzymes, the resulting fragment was ligated into a fragment of pSKPD25 digested with the same enzymes. The resulting plasmid was transformed into *E. coli *and designated as pSKPD253.

The chloramphenicol resistance gene was obtained by PCR amplification from plasmid pACYC184 using primers carrying a *Bam*HI(CmF-BamHI) and *Xba*I (CmR-XbaI) restriction site. The PCR product was digested with the two enzymes and cloned into pSKPD253 cut with the same enzymes. After ligation, the resulting plasmid was transformed into *E. coli*, verified by restriction analysis and designated as pSKPD25Cm3. The plasmid was digested with *Not*I and *Spe*I and the resulting fragment was ligated into pSS4245 which was doubly digested with the same enzymes. The resulting plasmid was designated as pSSP2D5Cm3 and transformed into *E. coli *SM10. Conjugation was conducted as described above by using Bp-WWD as the recipient *B. pertussis *strain with selection of Cm^R ^and Sm^S ^single colonies. The integration of *Cm^R ^*gene at its designated position was confirmed by PCR with the primers that specifically bind to only the upstream 5' (5'FPD2-int and 5'RCM-int primers), 3' (3'FCM-int and 3'RPD2-int primers) downstream flanking regions, and inside the *Cm^R ^*gene.

#### Integration of prn gene under control of fha promoter

The structural gene of PRN was amplified from *B. pertussis *DNA using a primer starting at the ATG start codon (F) and a primer carrying an *Xba*I (R) restriction site. The 2,808 bp amplified product containing only the coding region and the terminator was treated by an 'A' tailing protocol (Promega, USA). The resulting fragment was cloned into pGEM-T easy vector to obtain a plasmid designated as pGEM-TPRN which was verified by restriction analysis. In an initial workup to create a second copy of the PRN gene driven by the stronger FHA promoter, the FHA promoter was isolated from *B. pertussis *DNA by PCR amplification and inserted ahead of the PRN gene. The FHA promoter was amplified by primers carrying the *BamH*I (FHAproF-BamHI) and a polylinker containing *Nde*I-*Xba*I (FHAR-MCS). The purified product was cut with *Bam*HI and *Xba*I then the recovered DNA fragment was ligated into pSKPD253 cut with the same enzymes. The resulting plasmid designated as pSKPD253Fp was verified by restriction analysis. This plasmid was cut with *Nde*I and *Xba*I, then ligated with the PCR product of the *prn *gene which was amplified from pGEMTPRN by PRNF-NdeI and PRNR-XbaI primers and cut with the same enzymes. The resulting plasmid was designated as pSKPD25FpPRN3 (Figure 5B). The conjugative construct was obtained by digesting this plasmid with *Not*I and *Spe*I and ligation into pSS4245 digested with the same enzymes. The resulting plasmid was designated as pSSPD2FpPRN. This construct was inserted at the selected location of the Bp-WWD chromosome to replace the chloramphenicol resistance marker introduced using the usual allelic-exchange procedures and screening as described above.

#### Expression of prn gene under control of prn promoter

The PRN promoter was cloned by PCR amplification of the *B. pertussis *DNA using primers with the restriction sites *BamH*I (PrnProF-BamHI) and *Nde*I (PRNProR-NdeI). The plasmid pSKPD25FpPRN3 was cut with *BamH*I and *Nde*I to generate a fragment which had lost the FHA promoter. The PRN promoter was ligated in its place. After transformation into *E. coli *and verification by restriction analysis, the resulting plasmid was designated as pSKPD25PRN3 (Figure 5C). The plasmid was cut with *Not*I and inserted into pSS4245 cut with the same enzyme. The resulting construct, pSSPD2prn was transferred into *E. coli *SM10 to conduct the allelic exchange. The resulting *B. pertussis *strain was designated as Bp-WWE. Integration of the *prn *gene at its designated position was confirmed by PCR with the primers that specifically bind only to the upstream 5' (5'FPD2-int and PRNProR-NdeI primers), 3' (PRNF-int and 3'RPD2-int primers) downstream flanking regions, and inside the *prn *gene.

### PT, FHA and PRN expression in shake flask culture

The Bp-WWC, Bp-WWD and Bp-WWE strains were grown in shake flasks with 100 mL MSS medium supplemented with methylated β-cyclodextrin (1 g/l) at 35°C with shaking speed of 200 rpm. After 32-48 h of growth, the culture supernatants were collected and assayed by ELISA to quantify the PT and FHA expression level. As PRN releasing from its membrane-bound precursor is the result of an imprecise cleavage by unidentified proteases [34], PRN expression was determined by Western blot with densitometric analysis to evaluate the integrity of the antigen. This assay was conducted both on the clarified culture supernatant and the cell extract obtained by heating cell suspension in isotonic buffer (10 mM Tris-HCl pH 8.0, 150 mM NaCl, 0.002% NaN_3_, and 1 mM PMSF) at 60°C for 30 min and the supernatant was collected after centrifugation at 10,000 × g, 4°C for 30 min.

### ELISA assay for PT and FHA

Purified rabbit polyclonal antibodies against PT or FHA (NLAC, Thailand) with the dilution of 1:1000 in carbonate/bicarbonate buffer (pH 9.6) were coated in 96-well plates (NUNC Maxisorp, Denmark) for 100 μL per well and incubated overnight at 4°C. After 3 time-washing with phosphate-buffered saline pH 7.4 containing 0.1% Tween 20 (PBST), blocking was performed using 100 μL per well of 3% bovine serum albumin (BSA)-PBST then incubated at 37°C for 1 h. After discarding the blocking buffer and washing, dilutions of the standard PT, FHA or samples were loaded and incubated at 37°C for 1 h. Then, anti-PT mouse monoclonal antibody (Abcam, USA) at 1:30,000 dilution or anti-FHA mouse monoclonal antibody (NIBSC, UK) at 1:10,000 dilution in blocking buffer was added and incubated under the same conditions. After washing the wells for three times with PBST, 100 μL of rabbit anti-mouse (H + L) IgG-HRP conjugate (Abcam, USA) in blocking buffer at 1:10,000 dilution was used as secondary antibody and incubated for 37°C for 1 h. After washing with PBST, 100 μL of enzyme substrate, 3,3',5,5'-tetramethylbenzidine (KPL, USA), was added. The colour reaction was terminated with 1 N HCl, 100 μL per well. Optical density was measured at 450 nm using a microtiter plate reader. ELISA assay for PT and FHA of each recombinant strain was done in three replicates using three independent cultures.

### Western blot assay for PRN

Dilutions of standard PRN and samples were resolved in a 10% SDS-PAGE gel then transferred to a PVDF membrane using a semi-dry blotting system. The membrane was blocked with 5% skim milk in PBST for 1 h. After discarding the blocking solution, the membrane was incubated with 20 mL anti-PRN sheep serum (NIBSC, UK) at 1:10,000 dilution in blocking buffer for 1 h, then washed three times with PBST. The membrane was then incubated under the same conditions with 20 mL of rabbit anti-sheep IgG-HRP conjugate (Santa Cruz Biotechnology, USA) and washed again. The membrane was then immersed in 3,3'-diaminobenzamidine until the brown colour developed. The reaction was terminated by rinsing 2-3 times with de-ionized water, then left to dry at room temperature. Western blot of PRN of the three recombinant strains was performed in three replicates using cell extracts from three independent cultures of each strain. The membranes were scanned and converted to a picture file. PRN concentrations were derived by densitometric analysis of the sample and reference bands using ImageJ software http://rsbweb.nih.gov/ij/.

### Genetic stability

The strains were cultured in 100 mL MSS medium at 35°C and agitated at 200 rpm for 48 h, then 0.1 mL of culture was transferred into 100 mL MSS and incubated under the same conditions. This step was repeated four more times. Each transfer corresponded to 50 generations. The culture was diluted and plated on MSS agar. Thirty isolated colonies of a final plating were randomly picked and analysed by PCR to detect the expected presence of *ptx *and *prn *inserts.

### CHO cell-clustering assay

CHO cell clustering activity was determined by the method of Hewlett *et al. *[28] In short, CHO cells were cultured in the cRPMI 1640 medium supplemented with 10% fetal bovine serum. The cells were incubated at 37°C under 5% CO_2 _atmosphere. After trypsinization, 200 μL of CHO cell suspension at density of 2 × 10^4 ^cells/mL were seeded in a 96-well micro-culture plate. Test samples and reference PT toxin were serially diluted at ten-fold intervals in phosphate-buffered saline (PBS) pH 7.4 and a 25 μL volume of the dilutions was added to each well. After incubation for 48 h under the same conditions to permit maximal clustering, cells were stained with crystal violet and photographed.

## Abbreviation

PT: Pertussis toxin; PRN: Pertactin; FHA: Filamentous hemagglutinin; rPT: recombinant Pertussis toxin; ELISA: Enzyme-Linked Immunosorbent Assay

## Competing interests

The authors declare that they have no competing interests.

## Authors' contributions

WB, AL and PP conceived the study. WP, CB, AI and JP designed the experiments. WB wrote the draft of manuscript, JP and WP revised the manuscript. All authors read and approved the final version of the manuscript.
